# A prospective study of the relationships between change in body composition and cardiovascular risk factors across the menopause

**DOI:** 10.1097/GME.0000000000001721

**Published:** 2021-02-01

**Authors:** Aaron Dehghan, Senthil K. Vasan, Barbara A. Fielding, Fredrik Karpe

**Affiliations:** 1 Oxford Centre for Diabetes, Endocrinology and Metabolism, University of Oxford, Churchill Hospital, Oxford, UK; 2 Department of Nutritional Sciences, University of Surrey, Guildford, UK; 3 NIHR Oxford Biomedical Centre, Oxford University Hospital Trust and University of Oxford, Oxford, UK.

**Keywords:** Cardiovascular disease, DEXA, Fat distribution, Lipids, Menopause

## Abstract

**Objective:**

Menopause increases the risk of cardiovascular disease (CVD) which in part has been attributed to the rise in cholesterol and blood pressure (BP). This study examined the hypothesis that menopausal changes in body composition and regional fat depots relate to the change in CVD risk factors.

**Methods:**

A prospective recall study was designed to capture premenopausal women to be re-examined soon after menopause. A total of 97 women from the Oxford Biobank underwent dual x-ray absorptiometry, blood biochemistry, and BP readings pre- and postmenopause.

**Results:**

Despite minimal changes in body weight over the 5.1 ± 0.9 year follow-up period, there was an increase in total fat mass and a decline in lean mass, where the proportional change of regional fat mass was the greatest for the visceral fat depot (+22%, *P* < 0.01). Plasma ApoB (+12%, *P* < 0.01) and C-reactive protein (+45%, *P* < 0.01) increased as did systolic (+7%, *P* < 0.001) and diastolic BP (+5%, *P* < 0.001). Plasma nonesterified fatty acids decreased (−20%, *P* < 0.05) which may reflect on a change in adipose tissue function across the menopause. PCSK-9 decreased (−26%, *P* < 0.01) which suggests a compensation for the postmenopausal reduction in low-density lipoprotein receptor activity. Using multilinear regression analyses the changes in ApoB and diastolic BP were associated with visceral fat mass change, but this association was lost when adjusted for total fat mass change.

**Conclusion:**

The increase in CVD risk factor burden across menopause may not be driven by changes in body composition, rather by functional changes in end organs such as adipose tissue and liver.

Cardiovascular disease (CVD) is the leading cause of mortality in women.^1^ The incidence of CVD in women before 50 years of age appears to lag behind that of men by approximately 10 years.^2^ After the age of 50, which tends to coincide with the menopause transition, the sex differences in incidence rapidly level out.^3^ This is explained by an accelerated increase in the incidence of female CVD^1,2^ around menopause, with postmenopausal women having twice the rate of cardiovascular events compared with aged-matched premenopausal women.^4^ The increase seen across the menopausal transition is significantly higher than that seen at the same ages in men, suggesting menopause as a potential risk factor for CVD independent of aging.^5^

During menopausal transition, women show numerous metabolic changes, independent of aging, which could account for the rise in cardiovascular events associated with menopause. Menopause has been shown to be associated with a rise in blood pressure (BP) with both cross-sectional and longitudinal studies showing an increase in systolic and/or diastolic BP.^6^ However, in the longitudinal studies the findings are inconsistent with some studies showing no BP changes^7,8^ or even a reduction in BP in the years following the menopause.^9^ Other metabolic changes seen with the menopause include an adverse effect on the lipid profile and glucose metabolism^10,11^ including increments in apolipoprotein B (ApoB), triglycerides, total cholesterol, and low-density lipoprotein-cholesterol (LDL-C) across this period. Longitudinal studies have shown that changes in the lipid profile including LDL-C, ApoB, and triglycerides tend to primarily occur in the 2 years surrounding the final menstrual period.^5^ In some studies, these changes have stretched further into the early postmenopausal period (24 mo from the final menstrual period) and up to 6 years after.^12^

The menopause is also known to be associated with increases in total fat mass and changes in body fat distribution toward a more android phenotype.^10^ The majority of evidence to support these fat distribution changes come from cross sectional studies comparing premenopausal to postmenopausal women using dual energy X-ray absorptiometry (DEXA) measurements.^13,14^ However, fewer studies have examined longitudinally using computed tomography (CT) imaging of abdominal fat distribution alongside DEXA for other parameters.^15-17^ Although CT is well validated for this purpose a wider use is restricted by the radiation dose, variability in image analysis, and cost. In contrast, DEXA scans involve less radiation exposure, are less expensive, and are now validated against CT measurements to give accurate estimates of both visceral adipose tissue (VAT) and subcutaneous fat.^18^ We have recently shown in a large general population that DEXA-measured VAT and android fat masses are associated with a CVD and type 2 diabetes risk factors whereas the abdominal subcutaneous fat alongside gynoid and leg show protective associations,^19^ and the protective association is stronger in women.

The aims of this study were to prospectively measure changes in metabolic parameters and body fat distribution across the menopause including markers for nonesterified fatty acids (NEFA) and proprotein convertase subtilisin/kexin type (PCSK-9) reflecting metabolic tissue function. We then aimed to answer a specific question as to whether, within a large population-based cohort of apparently healthy British women, their individual changes in body fat depots were associated with the worsening of CVD risk factors seen across the same time period.

## METHODS

### Participants

We initially identified 150 White women aged between 44 and 48 years from the Oxford Biobank,^20^ who had undergone a DEXA scan and were premenopausal at their initial screening visit, and at subsequent follow-up were aged 50 years or above. Of these, a total of 103 women were successfully recalled for a second visit ranging between 3 and 7 years from their initial visit. To define menopausal status, we used the Stages of Reproductive Aging Workshop +10 staging criteria.^21^ Any women self-identifying as peri- or postmenopausal, based on symptoms and/or menstrual cycle, were included in the study. If the menopausal status was uncertain then FSH levels were used to determine their menopausal status and a level of >25 IU/L was used as a cutoff for inclusion into the postmenopausal group. We excluded two women who had an FSH <25 IU/L and four women who were on hormone therapy, leaving a total sample size of 97 women for analysis. All participants gave informed, written consent and the study was approved by the Oxfordshire Clinical Research Ethics Committee.

### Anthropometric and biochemical measurements

The pre and postmenopausal visits had identical measurements which included conventional anthropometric measurements (height, weight, and BMI), a DEXA scan, blood pressure, and a fasting venous blood sample using standard protocols by trained research nurses.^20^ Plasma was analyzed for triglycerides, total cholesterol, HDL-cholesterol (HDL-C), ApoB, NEFA, C-reactive protein (CRP), insulin, and glucose concentrations as previously described.^20^LDL-C levels were calculated using the Friedewald formula. Plasma FSH and PCSK-9 levels were determined using AlphaLISA (PerkinElmer, Waltham, MA) assays as per the manufacturer's protocols. The pre- and postmenopausal samples were assayed at different time points and different batches but were done using the same analytical platforms. Our assays undergo periodic quality assessment against national calibration standards (UK-NEQAS). The DEXA was performed on GE Lunar iDXA which included quantification of total body lean and fat mass and regional android, visceral, subcutaneous, gynoid, and leg fat and lean masses.^20^

### Statistical analysis

Data are presented as mean values ± standard deviations or when appropriate median + interquartile range. The measurements at the two time points were compared using paired *t* tests for normally distributed and log-transformed variables and Wilcoxon signed-rank test was used as appropriate.

The value for change (Δ-change) was calculated as the mean difference between pre- and postmenopausal measurements for clinical measurements, biochemical analyses, and body composition. To normalize any skewed data and allow comparison between the DXA and metabolic measurements, which are recorded in different units, all data (pre + postmenopausal values and delta values) were transformed into z-scores using Fisher Yates transformation. This allows direct comparison of magnitude of association per 1 SD change.^19^ To evaluate the correlation between body compartments, blood pressure, and the lipid profile, Pearson coefficients were estimated using the z-scores in both the pre- and postmenopausal groups.

Univariate and multivariate (adjusted for total fat mass, time between visits, premenopausal age, and baseline outcome value) linear regression models were used initially to examine the association of z-transformed premenopausal fat and lean compartments (independent variables) on the change in systolic blood pressure, diastolic blood pressure, and ApoB levels (dependent variables) across the menopause. Similar analyses (adjusting for same confounders as above) were used to examine the association of z-transformed changes in fat and lean compartments on z-transformed changes in systolic blood pressure, diastolic blood pressure, and ApoB levels across the menopause and results are presented as estimate (β) and 95% confidence intervals.

Statistical analysis was performed using SPSS Statistics version 24 (IBM, SPSS products, Chertsey, UK). Two-tailed *P* values of 0.05 or less were considered significant.

## RESULTS

### Changes in body composition, blood pressure, and lipid risk factors across menopause

**FIG. 1:**
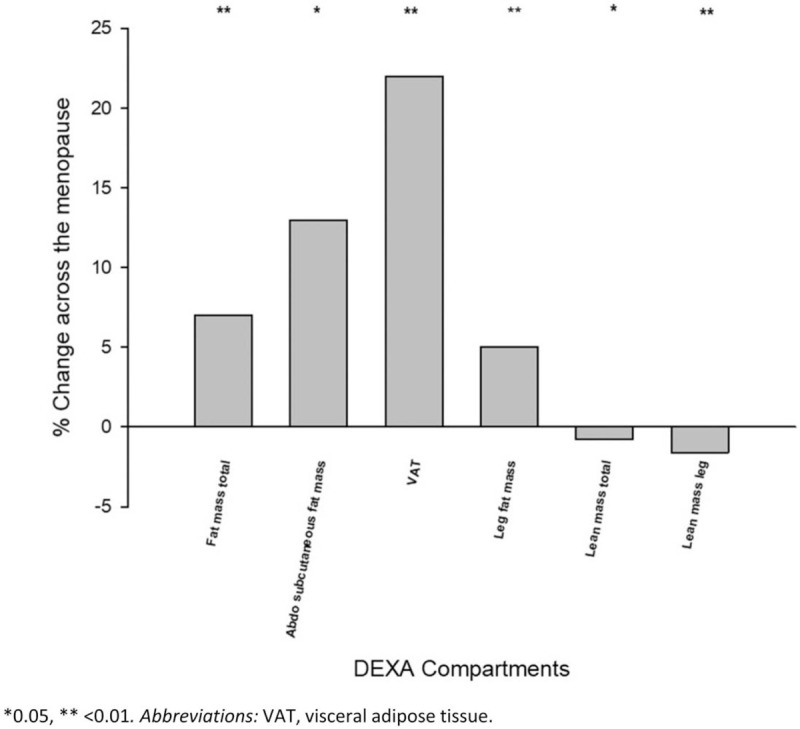
The percentage (%) change across the menopause of different fat depots and lean tissue measured by DEXA. DEXA, dual energy X-ray absorptiometry.

There were 5.1 ± 0.9 years between the pre and postmenopausal visits in the 97 women in this study (Table 1). In this time period, the overall change in body weight was not statistically significant but there were significant changes in body composition. The total fat mass increased by 1.3 kg (*P* < 0.01); of the fat depots measured, the major contributor to this was leg fat (0.4 kg, *P* < 0.01). The total lean mass decreased by 0.4 kg (*P* < 0.05), including a reduction in skeletal muscle mass of 0.2 kg (*P* < 0.01) measured as the (appendicular) lean mass of the legs. The relative increase in different fat depots varied with the lowest being leg fat mass at 5% (*P* < 0.01), followed by subcutaneous abdominal fat at 13% (*P* < 0.05), and lastly the greatest proportional increase being in VAT mass at 22% (*P* < 0.01) (Fig. 1).

**TABLE 1:** Clinical characteristics of the study population

*N* = 97	Premenopausal	Postmenopausal	Δ-change
*Demographics*			
Age (y)	46.7 (1.2)	51.8 (0.9)	5.1 (0.9)^a^
*Anthropometry*			
Height (cm)	165.5 (6.4)	165.2 (6.6)	–0.3 (1.3)^b^
Weight (kg)^c^	66.4 (59.6, 73.2)	67.0 (60.6, 73.4)	0.5 (4.8)
BMI (kg/m^2^)	25.4 (0.5)	25.1 (0.4)	0.30 (1.9)
*Metabolic traits*			
Systolic blood pressure (mm Hg)	116 (13)	123 (14)	7.2 (13.0)^a^
Diastolic blood pressure (mm Hg)	73 (9)	76 (9)	3.2 (7.7)^a^
CRP (mg/L)^c^	0.30 (–0.04, 0.64)	0.38 (–0.15, 0.91)	0.4 (1.8)^d^
Glucose (mmol/L)^e^	5.1 (4.9, 5.4)	5.2 (4.9, 5.5)	0.05 (0.48)
Insulin (mU/L)^e^	11.8 (9.3, 14.3)	10.6 (7.9, 13.2)	0.7 (15.15)
NEFA (μmol/L)^c^	475 (322.6, 591.2)	415 (242.3, 569.0)	–60 (264)^a^
Triglyceride (mmol/L)^c^	0.79 (0.59, 1.00)	0.80 (0.58, 1.02)	0.06 (0.45)
Total cholesterol (mmol/L)	5.25 (0.98)	5.36 (1.01)	0.1 (0.79)
HDL cholesterol (mmol/L)	1.60 (0.41)	1.58 (0.46)	–0.02 (0.36)
LDL cholesterol (mmol/L)	3.23 (0.83)	3.33 (0.86)	0.1 (0.71)
ApoB (g/L)	0.90 (0.22)	1.00 (0.24)	0.1 (0.14)^a^
PCSK-9 (ng/mL)	452 (292, 612)	369 (196, 542)	–112.5 (456.7)^d^
FSH (IU/L)^e^	10.0 (8.0, 12.0)	49.0 (14.0, 85.0)	38.4 (39.5)^a^
*DEXA body composition*			
Fat mass total (kg)^c^	21.4 (16.5, 26.2)	23.1 (18.3, 27.9)	1.3 (4.28)^d^
Abdominal Subcutaneous fat mass (kg)	1.3 (0.66)	1.4 (0.69)	0.1 (0.36)^b^
Android fat mass (kg)^c^	1.6 (1.0, 2.1)	1.6 (1.0, 2.2)	0.1 (0.51)^b^
Visceral adipose tissue (kg)^e^	0.29 (0.12, 0.46)	0.31 (0.04, 0.58)	0.06 (0.21)^d^
Leg fat mass (kg)^c^	7.8 (6.2, 9.5)	8.5 (6.7, 10.3)	0.4 (1.32)^d^
Lean mass total (kg)	41.9 (4.7)	41.5 (4.6)	–0.4 (1.46)^b^
Lean mass legs (kg)	14.1 (2.0)	13.8 (2.0)	–0.2 (0.71)^d^

Values are presented as mean + SD for parametric data and median + IQR for nonparametric. Delta (Δ) value presented with SD. ApoB indicates apolipoprotein B; BMI, body mass index; CRP, C-reactive protein; DEXA, dual energy X-ray absorptiometry; FSH, follicle-stimulating hormone; HDL, high-density lipoprotein; LDL, low-density lipoprotein; NEFA, nonesterified fatty acids; PCSK-9, proprotein convertase subtilisin/kexin type.

a *P* < 0.001.

b *P* < 0.05.

c Log-transformed for analysis and back-transformed for presentation.

d *P* < 0.01.

e Nonparametric Wilcoxon test otherwise paired *t* test used. *P* values represent comparison of mean/median difference between postmenopausal and premenopausal variables.

Both systolic and diastolic BP increased substantially across the menopause (Table 1). The changes of conventional lipids were small (Table 1): total cholesterol and triglycerides were largely unchanged together with HDL-C whereas there was a nonsignificant rise in LDL-C (+5%, *P* = 0.16). A significant increase in ApoB (+12%, *P* < 0.001), in the absence of changes in triglycerides, reflects increase in the LDL particle concentration in plasma across menopause. In terms of regulators of LDL particle numbers in plasma, lower plasma PCSK9 concentrations (−21%, *P* < 0.01) were observed after the menopause transition. There were no changes in fasting plasma glucose and insulin concentrations. There was however a reduction (−20%, *P* < 0.05) in plasma NEFA. There was a small but statistically significant increase in the plasma concentration of CRP (*P* < 0.01).

### Correlations between DEXA body compartments and BP and lipid CVD risk factors

Total and regional abdominal fat masses showed positive associations with BP and ApoB in both pre- and postmenopausal state (Suppl. Table 1 and 2). The associations were weak or absent with LDL-C but robustly negative with HDL-C. The associations between leg fat mass and ApoB and LDL-C were weak and statistically nonsignificant.

### Associations between the premenopausal DEXA body compartments and the change in BP and ApoB across the menopause

The premenopausal fat or lean mass compartment did not correlate with BP or ApoB in an unadjusted model, whereas adjustment for total fat mass brought out significant negative association between VAT and systolic BP and positive association between subcutaneous fat and diastolic BP suggesting that regional fat masses rather than total fat mass are drivers for these CVD risk factors (Table 2).

**TABLE 2:** Linear regression analyses (unadjusted and adjusted) using premenopausal z-DEXA body composition as the independent variable and z-transformed within woman change (postmenopausal − premenopausal value, z-Δ) traits

	z- Δ SBP		z-Δ DBP		z-Δ ApoB	
	β (95% CI)	*P* value	β (95% CI)	*P* value	β (95% CI)	*P* value
Unadjusted model						
*z-Fat mass*						
z-Android	0.08 (–0.13 to 0.28)	0.45	0.08 (–0.13 to 0.28)	0.46	0.09 (–0.12 to 0.29)	0.37
z- Subcutaneous	0.15 (–0.06 to 0.34)	0.15	0.14 (–0.06 to 0.34)	0.16	0.09 (–0.11 to 0.29)	0.39
z-VAT	–0.08 (–0.29 to 0.12)	0.39	–0.08 (–0.28 to 0.12)	0.43	0.07 (–0.13 to 0.28)	0.47
z-Leg	0.19 (–0.01 to 0.39)	0.07	0.11 (–0.09 to 0.31)	0.29	0.05 (–0.15 to 0.25)	0.62
z-Total fat mass	0.12 (–0.08 to 0.32)	0.25	0.07 (–0.12 to 0.27)	0.47	0.08 (–0.12 to 0.29)	0.42
*z-Lean mass*						
z-Leg	–0.04 (–0.24 to 0.17)	0.69	0.09 (–0.11 to 0.29)	0.40	0.06 (–0.15 to 0.26)	0.58
z-Total lean	–0.06 (–0.26 to 0.14)	0.59	0.08 (-0.12 to 0.29)	0.47	0.03 (–0.18 to 0.23)	0.81
Adjusted model^a^						
*z-Fat mass*						
z-Android	–0.25 (–0.86 to 0.35)	0.41	0.22 (–0.37 to 0.82)	0.45	0.22 (–0.42 to 0.87)	0.49
z-Subcutaneous	0.33 (–0.27 to 0.92)	0.28	0.72 (0.16 to 1.28)	0.013	0.08 (–0.55 to 0.71)	0.80
z-VAT	–0.39 (–0.67 to –0.11)	0.007	–0.25 (–0.52 to 0.03)	0.09	0.11 (–0.19 to 0.42)	0.47
z-Leg	0.16 (–0.23 to 0.56)	0.42	–0.07 (–0.46 to 0.33)	0.74	–0.16 (–0.57 to 0.26)	0.45
*z-Lean mass*						
z-Leg	–0.72 (–0.29 to 0.14)	0.51	0.04 (–0.17 to 0.25)	0.69	–0.02 (–0.24 to 0.21)	0.89
z-Total lean	–0.05 (–0.25 to 0.16)	0.66	0.06 (–0.14 to 0.25)	0.58	–0.06 (–0.27 to 0.16)	0.61

ApoB indicates apolipoprotein B; DBP, diastolic blood pressure; SBP, systolic blood pressure; VAT, visceral adipose tissue; DEXA, dual energy X-ray absorptiometry.

a Adjusted for premenopausal total fat mass, time between visits, premenopausal age, and baseline outcome value. B-estimate represents the SD Δ (postmenopausal − premenopausal) change in the outcome variable corresponding to a 1 SD change in the premenopausal fat depots.

### Associations between change in DEXA body compartments and change in BP and ApoB across menopause

To analyze whether parameters changing across menopause did so in a coordinated way we selected the established CVD risk factors that showed a robust increase across menopause, as above, and examined the relationship between the change of these factors with the change of the total and regional fat and lean masses.

Whereas the absolute change in systolic BP did not correlate significantly with change in total fat, regional fat, and lean masses across menopause, the rise in diastolic BP was significantly associated with the absolute increments in android and VAT fat masses. The decrease in lean mass across menopause was also unrelated to the increase in diastolic BP. The increase of ApoB across menopause was associated with increase in android, VAT, and total fat mass, but not with change in leg fat mass or lean mass.

We further investigated if the changes in the regional fat mass were independent of the total fat mass change, premenopausal age, time between visits, and baseline outcome value (Table 3). Following adjustments for the above confounders, the relationship between the increase in diastolic BP and android fat remained significant, but other associations were lost. Similarly, the relationships seen between regional fat compartments and ApoB levels were also lost when adjusted for change in total fat mass suggesting that any of the changes attributed to the regional compartments in the adjusted model are probably mediated by changes in total fat mass across the menopause.

**TABLE 3:** Linear regression analyses (unadjusted and adjusted) using z-transformed within woman change (postmenopausal − premenopausal value) = Δ for DEXA body composition as the independent variable and the Δ of systolic blood pressure, diastolic blood pressure, and ApoB as the dependent variable

	z-Δ SBP		z-Δ DBP		z-Δ ApoB	
	β (95% CI)	*P* value	β (95% CI)	*P* value	β (95% CI)	*P* value
Unadjusted model						
*z-Δ Fat mass*						
z-Δ Android	0.10 (–0.10 to 0.31)	0.32	0.22 (0.20 to 0.42)	0.031	0.24 (0.04 to 0.43)	0.020
z-ΔSubcutaneous	0.03 (–0.17 to 0.24)	0.72	0.13 (–0.06 to 0.33)	0.20	0.17 (–0.03 to 0.37)	0.10
z-Δ VAT	0.12 (–0.09 to 0.32)	0.25	0.22 (0.03 to 0.42)	0.027	0.21 (0.01 to 0.42)	0.035
z-Δ Leg	0.12 (–0.08 to 0.32)	0.24	0.12 (–0.08 to 0.32)	0.24	0.18 (–0.02 to 0.37)	0.08
z-Δ Total fat mass	0.11 (–0.09 to 0.31)	0.27	0.18 (–0.02 to 0.38)	0.08	0.24 (0.03 to 0.43)	0.020
*z-Δ Lean mass*						
z-Δ Leg	0.13 (–0.08 to 0.32)	0.22	0.10 (–0.11 to 0.30)	0.34	–0.03 (–0.23 to 0.18)	0.78
z-Δ Total lean	0.15 (–0.06 to 0.35)	0.15	0.09 (–0.11 to 0.29)	0.38	–0.05 (–0.25 to 0.16)	0.64
Adjusted model^a^						
*z-Δ Fat mass*						
z-Δ Android	0.25 (–0.51 to 1.02)	0.51	0.88 (0.16 to 1.60)	0.017	–0.11 (–0.88 to 0.65)	0.77
z-Δ Subcutaneous	–0.16 (–0.54 to 0.22)	0.41	–0.07 (–0.43 to 0.30)	0.72	–0.22 (–0.61 to 0.16)	0.24
z-Δ VAT	0.13 (–0.18 to 0.43)	0.41	0.23 (–0.05 to 0.53)	0.11	0.06 (–0.25 to 0.37)	0.71
z-Δ Leg	0.08 (–0.38 to 0.53)	0.74	–0.25 (–0.69 to 0.19)	0.26	–0.19 (–0.65 to 0.28)	0.43
*z-Δ Lean mass*						
z-Δ Leg	–0.12 (–0.09 to 0.32)	0.26	0.13 (–0.07 to 0.33)	0.21	–0.07 (–0.27 to 0.14)	0.51
z-Δ Total Lean	0.14 (–0.07 to 0.35)	0.19	0.11 (–0.09 to 0.31)	0.27	–0.08 (–0.29 to 0.14)	0.46

ApoB indicates apolipoprotein B; DBP, diastolic blood pressure; SBP, systolic blood pressure; VAT, visceral adipose tissue; DEXA, dual energy X-ray absorptiometry.

a Adjusted for z-Δ-total fat mass, time between visits, premenopausal age, and baseline outcome value. B-estimate represents the SD Δ (postmenopausal − premenopausal) change in the outcome variable corresponding to a 1 SD z-transformed Δ (postmenopausal − premenopausal fat depots).

## DISCUSSION

This study was designed to examine the longitudinal associations between body composition (fat and lean mass) with known CVD risk factors across the menopause transition. Despite a small and statistically nonsignificant increase in body weight there was an increase in total fat mass seemingly counterbalanced by a decrease in lean mass which appears to be driven by a reduction in skeletal muscle mass since the appendicular (leg lean mass) was proportionally most reduced. Greatest proportional increase across the menopausal transition was observed with VAT. These findings are in agreement with previous longitudinal CT-based studies^15-17^ and larger cross-sectional studies.^13,14^

There was an expected rise in ApoB without statistically significant changes in total cholesterol, triglycerides, HDL-C, and calculated-LDL, which aligns with previous studies showing that the pre to postmenopausal transition is associated with raised LDL-C and that postmenopausal women tend to have a predominance of small, dense LDL compared with premenopausal.^22-24^ This could add to an increased CVD risk beyond the measurement of conventional lipoproteins.^22-24^

Although the menopausal rise in ApoB was associated with change in visceral and abdominal fat mass, it was also associated with the change in total fat mass. When, however, the associations for regional fat depots were adjusted for other potential confounders there was no statistically significant association suggesting that the rise in ApoB is not independently associated with fat distribution changes across menopause. It is possible that other factors, presumably operating in the liver like LDL receptor activity, are likely to determine the ApoB concentration. The modest rise in ApoB with a largely unchanged LDL-C as we observe may relate to the decline in estrogen levels associated with menopause. Estrogen suppresses hepatic lipase that is involved in the transitioning of intermediate to LDLs^25^ and therefore an increase in its activity through the menopause is a possible explanation.

To our knowledge this is the first study to report longitudinal changes in PCSK-9 across the menopause and our findings contrast with previous cross-sectional studies that have found the opposite association comparing groups of pre and postmenopausal women.^26,27^ This reduction in PCSK-9 may signal a liver-specific adaptation to menopause with possible implications for hepatic cholesterol homeostasis and is likely to be related to pre- to postmenopausal partitioning of hepatic lipids leading to distinct patterns of lipoprotein secretions.^28^ A significant reduction in the fasting plasma NEFA concentration across menopause may implicate a menopause-related functional change in the control of adipose tissue lipolysis and therefore indirectly also in fat storage. It is noted that the drop in fasting NEFA across menopause is of the same order of magnitude as difference between the premenopausal female NEFA concentrations compared with age-equivalent men.^20^ It could be that menopause alters the tissue response away from a particularly efficient fat storing organ as in the premenopausal state^29-31^ to the male fat storage (android fat accumulation) pattern. It has also been shown that longitudinally women transitioning from premenopause to peri/post menopause have a decrease in subcutaneous adipose tissue lipolysis, although this was thought to be related to aging rather than menopause.^32^ These suggest that impaired adipose tissue and liver function across menopause could contribute to worsening of the CVD risk factor profile.

When examining the remaining biochemical tests, we found no changes in fasting glucose or insulin levels, which is consistent with existing data showing that the menopause transition does not negatively affect glucose metabolism.^7,33,34^ As expected, we also found that CRP increased across the menopause transition which is a well-recognized inflammatory risk marker for CVD in postmenopausal women.^35^

Previous studies across the menopause have shown inconsistent effect of menopause on systolic and diastolic BP.^6^ This could possibly relate to differing study designs, sample size, ethnic background, age-range of participants, menopause classification, years of follow-up, and different methods of BP monitoring as well as inclusion of both natural and surgically induced menopausal women, who have differing hormonal and cardiovascular outcomes.^36^ Change in android fat across menopause with an increase in diastolic BP is consistent with other observations that intrabdominal fat accumulation could be an important risk factor for high BP.^37^ Although not directly investigated in this study the most probable explanation for the rise in BP seen independent of fat accumulation is the rapid decline in endothelial vasodilator function related to estrogen deficiency across the menopause transition.^38^ Again, it seems the menopausal effect on blood pressure is only modestly linked to a change in fat mass and more likely regulated at the end organ, ie, vascular bed.

We recognize some of the strengths and limitations of our study. The strength is the longitudinal design of the study that included homogeneous, apparently healthy, White women, with a well-defined menopausal follow-up period and identical measurements taken at both time points using the same platforms thus eliminating variability in measurements. Although apparently healthy, due to nonavailability of data, we have not accounted for lifestyle factors such as diet, exercise, alcohol intake, or smoking status which can clearly impact metabolic health. Our results are also limited to the UK population and therefore generalizability of our finding among other ethnic groups is limited, due to inherent differences in body composition. The protocol involved re-examination of women within only ∼5y gap. It could well be that some anthropometric adaptations associated with menopause take longer to have their full effect on metabolic homeostasis.

## CONCLUSION

Our study provides evidence that across the menopause transition there are changes seen in body fat distribution toward a more android-like distribution alongside a worsening in cardiovascular risk factors across this same time period. However, in this study it seems that the changes in established CVD risk factors across the menopause are not attributed to any significant degree to body composition changes, or perhaps in particular the distinct increase in VAT. Although leg fat mass has been shown to have a protective association on CVD risk markers, the increase across menopause observed in this study was in line with a total fat mass increase and without a redistribution toward gynoid adiposity, any positive association of leg fat mass may have been counterbalanced by increments in other fat depots. We also observed a number of effects suggesting that qualitative and functional changes in metabolic organs occur across menopause and these effects might have a stronger impact on CVD risk profile than changes in body composition.

## Supplementary Material

Supplemental Digital Content
