# Supplementary material for: A prospective study of the relationships between change in body composition and cardiovascular risk factors across the menopause
Source: Menopause. 2021 Feb 1;28(4):400–6. doi: 10.1097/GME.0000000000001721 (PMC8284369; doi:10.1097/GME.0000000000001721)
Supplement: Supplemental Digital Content [file menop-28-400-s001.docx]

Supplementary information

|  |  | **Pre-menopausal** | | | | | |
| --- | --- | --- | --- | --- | --- | --- | --- |
|  |  | z-Systolic Blood Pressure | z-Diastolic Blood Pressure | z-Total cholesterol | z-LDL  cholesterol | z-HDL cholesterol | z-ApoB |
| **Pre-menopausal values** | z-Fat Mass Android | 0.27^a^ | 0.40^b^ | 0.11 | 0.23^c^ | -0.45^b^ | 0.35^b^ |
|  | z-abdominal subcutaneous Fat mass | 0.24^c^ | 0.38^b^ | 0.08 | 0.20 | -0.42^b^ | 0.31^a^ |
|  | z-Visceral Adipose Tissue | 0.22^c^ | 0.33^b^ | 0.11 | 0.21^a^ | -0.46^b^ | 0.35^b^ |
|  | z-Fat mass legs | 0.11 | 0.20 | -0.06 | 0.08 | -0.36^b^ | 0.17 |
|  | z-Fat Mass total | 0.25^c^ | 0.38^b^ | 0.06 | 0.19 | -0.44^b^ | 0.30^a^ |
|  | z-Lean mass legs | 0.28^a^ | 0.21^c^ | -0.03 | -0.01 | -0.11 | 0.05 |
|  | z-Lean mass total | 0.31^a^ | 0.23^c^ | -.03 | -0.03 | -0.05 | 0.002 |

^c^ P < .05, ^a^ P < .01, ^b^ P < .001 otherwise not significant P > 0.05. Abbreviations: LDL, Low density lipoprotein; HDL, High density lipoprotein; ApoB, Apolipoprotein B.

Supplement Table 1. Pearson correlation coefficients (r) matrix between pre-menopausal DEXA body compositions against pre-menopausal CVD risk factors, using Z standardised scores.

|  |  | **Post-menopausal** | | | | | |  |
| --- | --- | --- | --- | --- | --- | --- | --- | --- |
|  |  | z-Systolic Blood Pressure | z-Diastolic Blood Pressure | z-Total cholesterol | z-LDL  cholesterol | z-HDL cholesterol | z-ApoB | |
| **Post-menopausal values** | z-Fat Mass Android | 0.26^a^ | 0.43^b^ | 0.15 | 0.22^a^ | -0.42^b^ | 0.36^b^ | |
|  | z-abdominal Subcut Fat mass | 0.27^c^ | 0.43^b^ | 0.11 | 0.19 | -0.41^b^ | 0.33^b^ | |
|  | z-Visceral Adipose Tissue | 0.26^a^ | 0.35^b^ | 0.10 | 0.13 | -0.38^b^ | 0.26^c^ | |
|  | z-Fat mass legs | 0.21^a^ | 0.21^a^ | 0.01 | 0.06 | -0.32^c^ | 0.16 | |
|  | z-Fat Mass total | 0.27^c^ | 0.37^b^ | 0.09 | 0.17 | -0.42^b^ | 0.30^c^ | |
|  | z-Lean mass legs | 0.15 | 0.26^a^ | -0.03 | 0.01 | -0.29^c^ | 0.07 | |
|  | z-Lean mass total | 0.17 | 0.23^a^ | -0.05 | -0.03 | -0.21^a^ | 0.02 | |

^a^ P < .05, ^c^ P < .01, ^b^ P < .001 otherwise not significant P > 0.05. Abbreviations: LDL, Low density lipoprotein; HDL, High density lipoprotein; ApoB, Apolipoprotein B.

Supplement Table 2. Pearson correlation coefficients (r) matrix between post-menopausal DEXA body compositions against post-menopausal CVD risk factors, using Z standardised scores.
